# Arthroscopic repair of knee cartilage defects with minced cartilage autograft: a 6-month follow-up case report study

**DOI:** 10.1093/jscr/rjad260

**Published:** 2023-06-20

**Authors:** Konstantin Mitev

**Affiliations:** Orthopedic and Traumatology Department, Zan Mitrev Clinic, Skopje, Republic of North Macedonia

## Abstract

Knee cartilage defects can cause significant pain and disability, leading to progressive degenerative changes if left untreated. Arthroscopic repair of these defects with minced cartilage autograft has emerged as a promising alternative to traditional surgical techniques. Articular cartilage was harvested from the edge of the osteochondral defect and from the edge of the supracondylar notch with a shaver. After that, the mixed cartilage was combined with prp in thrombinator were aplicated on the defect covered with fibrin glue. Postoperatively, the patient reported significant improvement in pain and function.

## INTRODUCTION

Knee cartilage defects can cause significant pain and disability, leading to progressive degenerative changes if left untreated [1]. Arthroscopic repair of these defects with minced cartilage autograft (MCA) has emerged as a promising alternative to traditional surgical techniques [2, 3]. This case report study aims to evaluate the efficacy of MCA in the repair of knee cartilage defects, with a 6-month follow-up.

## CASE REPORT

**Figure 1:**
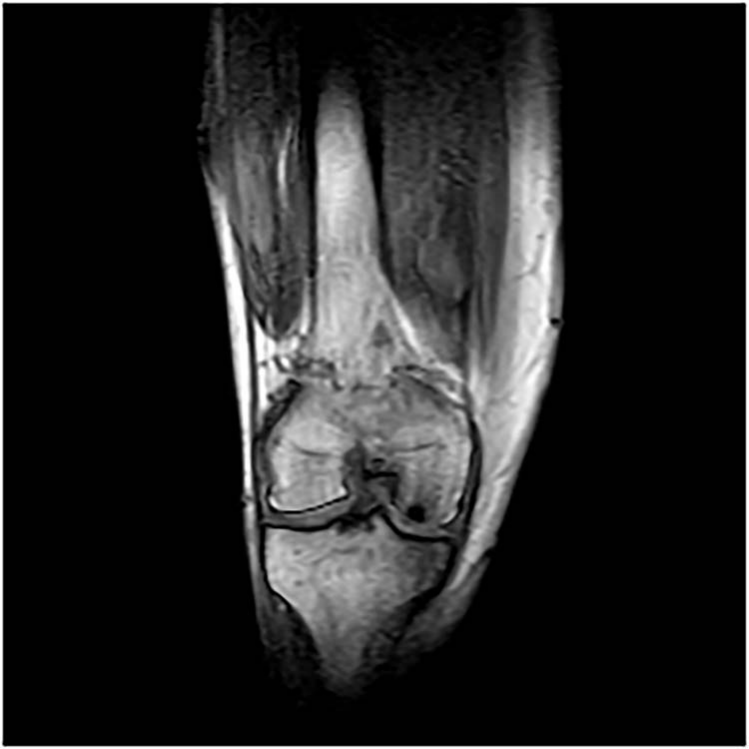
MRI images—osteochondral defect of the knee.

**Figure 2:**
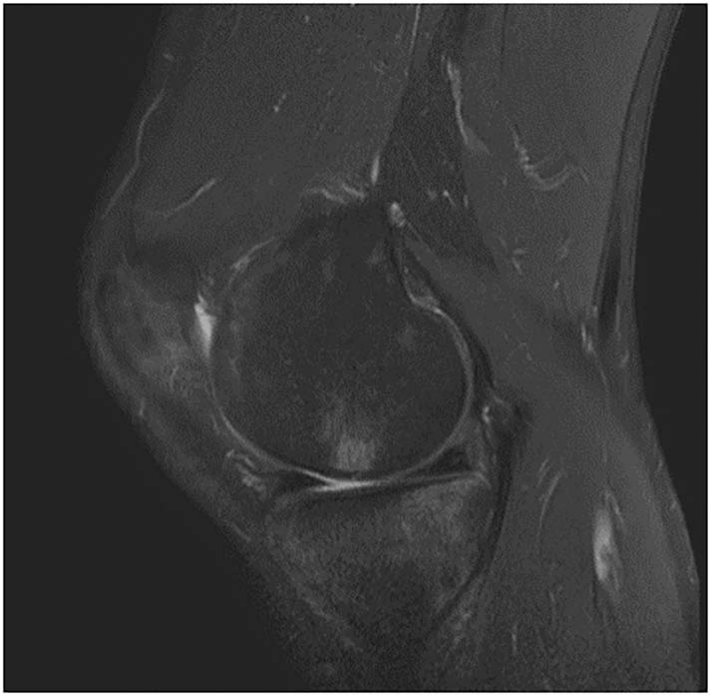
MRI images—osteochondral defect of the knee.

A 55-year-old male patient with a history of knee pain and difficulty with weight-bearing activities on the right side underwent arthroscopic repair of a large cartilage defect in his knee with MCA derived from his own knee cartilage (Figs 1, 2).

## MATERIAL AND METHOD

**Figure 3:**
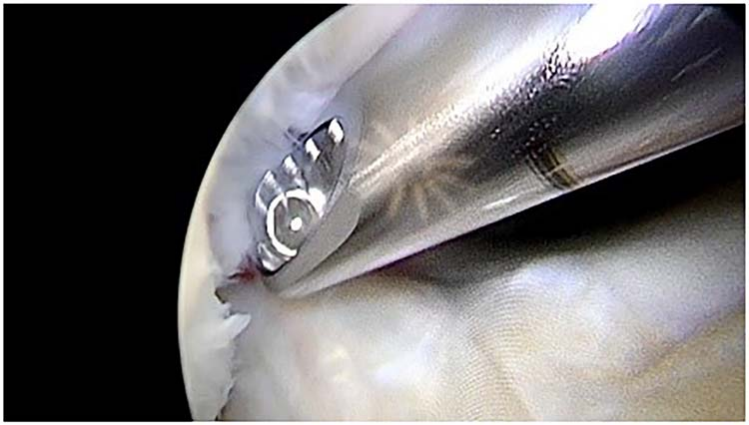
Shaver debridement.

**Figure 4:**
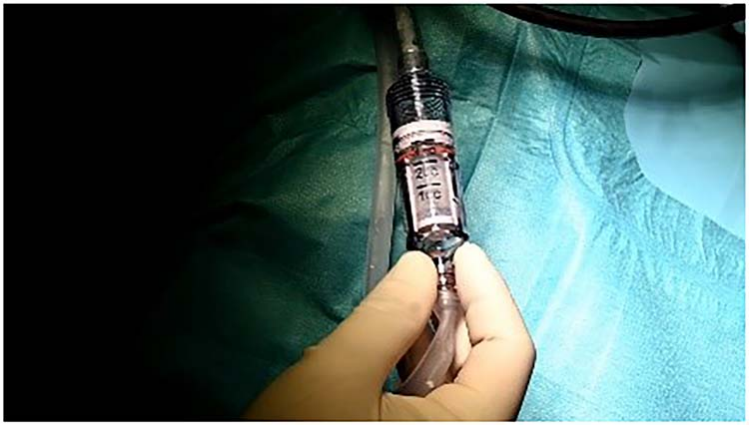
Mixed cartilage.

**Figure 5:**
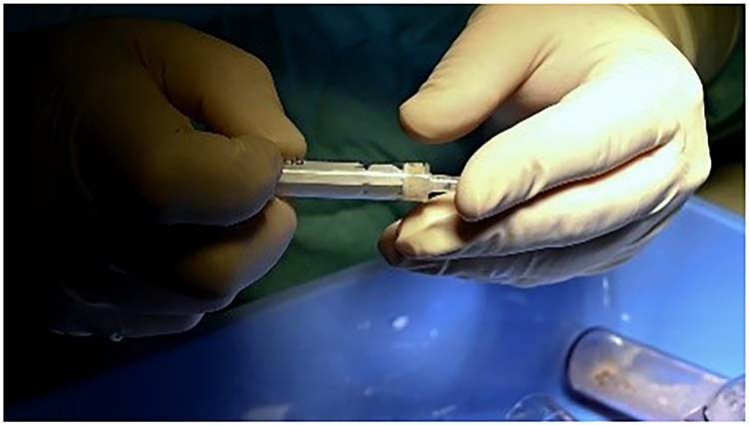
Mixed cartilage.

**Figure 6:**
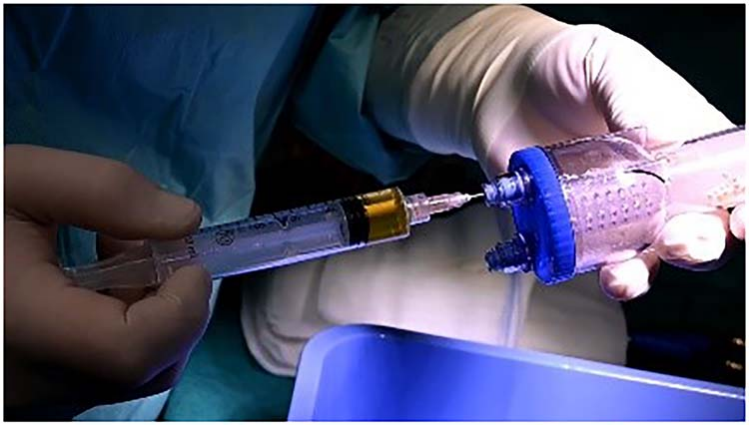
Prp in thrombinator.

**Figure 7:**
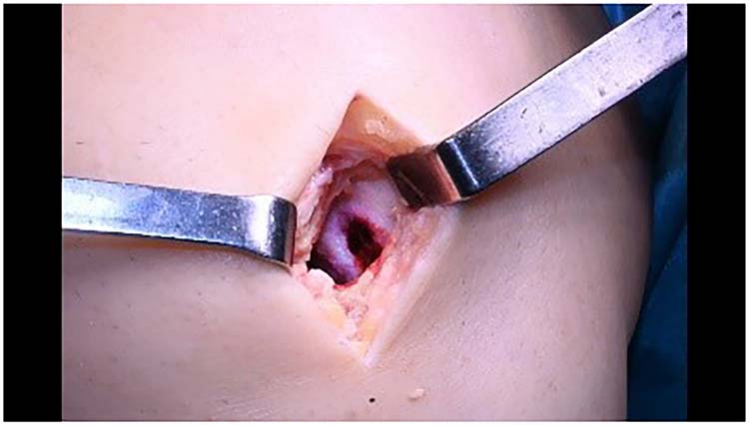
Mini open arthrotomy.

**Figure 8:**
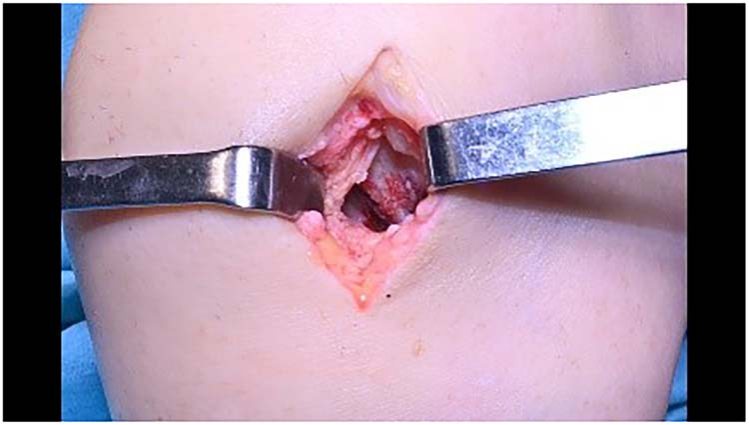
Cartilage application with fibrin glue.

Articular cartilage was harvested from the edge of the osteochondral defect and from the edge of the supracondylar notch with a shaver 4.0 (Fig. 3). After that, the mixed cartilage combined with prp in thrombinator were aplicated on the defect covered with fibrin glue (Figs 4–8).

**Figure 9:**
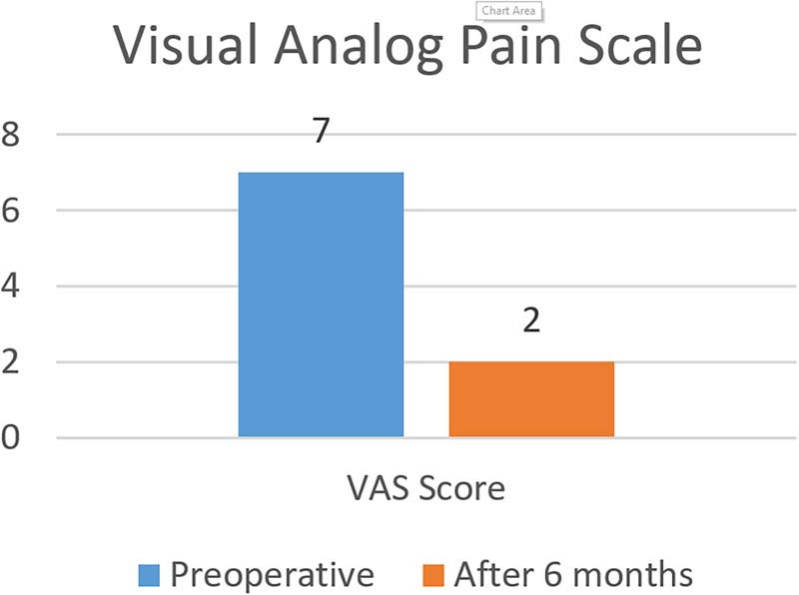
Pre and postoperative visual analog pain scale.

**Figure 10:**
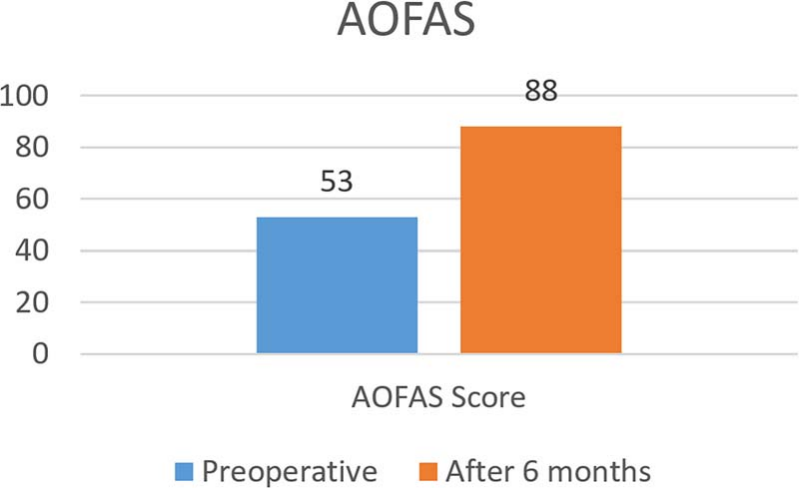
Pre and postoperative AOFAS score.

Postoperatively, the patient reported significant improvement in pain and function. Clinical evaluation, including the International Knee Documentation Committee (IKDC) score and the visual analog scale (VAS) for pain, was performed at 6 months after the procedure. The VAS score improved from 7 preoperatively to 2 at 6 months, whereas the IKDC score improved from 53 preoperatively to 88 at 6 months (Figs 9 and 10). No major complications were reported.

## DISCUSSION

This case report study supports the efficacy of MCA in the repair of knee cartilage defects. The results suggest that this technique may be a safe and effective alternative to traditional surgical options, offering significant improvement in pain and function for patients with knee cartilage defects. The use of arthroscopy in this procedure allows for a minimally invasive approach, with the potential for faster rehabilitation and a lower risk of complications compared with open surgical techniques.

Further randomized controlled trials with larger sample sizes and longer follow-up periods are needed to confirm these findings and to fully understand the potential benefits and limitations of this technique.

## CONCLUSION

The results of this case report study suggest that MCA may be a promising option for the repair of knee cartilage defects. This approach, combined with arthroscopy, may offer significant improvement in pain and function for patients with these injuries, with a low risk of major complications. Further research is needed to validate these findings and to fully understand the potential benefits and limitations of this technique.

## CONFLICT OF INTEREST STATEMENT

None declared.

## FUNDING

None.
